# Amyloidogenic Peptides in Human Neuro-Degenerative Diseases and in Microorganisms: A Sorrow Shared Is a Sorrow Halved?

**DOI:** 10.3390/molecules25040925

**Published:** 2020-02-19

**Authors:** Kristina Endres

**Affiliations:** Department of Psychiatry and Psychotherapy, University Medical Center Johannes Gutenberg-University Mainz, 55131 Mainz, Germany; Kristina.endres@unimedizin-mainz.de; Tel.: +49-6131-17-2133

**Keywords:** amyloid, Alzheimer’s disease, biofilm, neurodegeneration, microbiota, Parkinson’s disease

## Abstract

The term “amyloid” refers to proteinaceous deposits of peptides that might be generated from larger precursor proteins e.g., by proteolysis. Common to these peptides is a stable cross-β dominated secondary structure which allows self-assembly, leading to insoluble oligomers and lastly to fibrils. These highly ordered protein aggregates have been, for a long time, mainly associated with human neurodegenerative diseases such as Alzheimer’s disease (Amyloid-β peptides). However, they also exert physiological functions such as in release of deposited hormones in human beings. In the light of the rediscovery of our microbial commensals as important companions in health and disease, the fact that microbes also possess amyloidogenic peptides is intriguing. Transmission of amyloids by iatrogenic means or by consumption of contaminated meat from diseased animals is a well-known fact. What if also our microbial commensals might drive human amyloidosis or suffer from our aggregated amyloids? Moreover, as the microbial amyloids are evolutionarily older, we might learn from these organisms how to cope with the sword of Damocles forged of endogenous, potentially toxic peptides. This review summarizes knowledge about the interplay between human amyloids involved in neurodegenerative diseases and microbial amyloids.

## 1. Introduction

Amyloidogenic peptides or proteins are well known to be the main culprit of various human diseases, but more recently, several of these proteins with physiological function have been identified also in humans (reviewed in [1]). One prominent example is the pigment cell-specific pre-melanosomal protein (PMEL). Fibrils of this protein might offer a scaffold for highly reactive melanin intermediates and thereby protect the synthesizing cells [2]. A second example of functional amyloids is the RIP1/RIP3 necrosome signaling complex [3]. Interestingly, RIP1/RIP3 seem to mediate the necroptosis of dopaminergic neurons in a murine Parkinson’s disease (PD) model [4] and PMEL amyloid formation is based on cleavage by BACE-2 and γ-secretase [2], both enzymes also capable of cleaving the Alzheimer’s disease (AD)-relevant amyloid precursor protein (APP).

**Figure 1:**
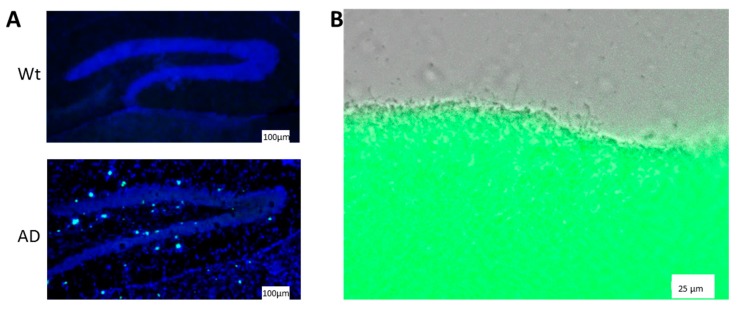
Amyloid deposits in the brain of an AD mouse model and in *Escherichia coli* biofilm layer. (**A**) Sagittal sections of murine brain were fixed with 4% PFA and amyloid plaques stained with ThT (turquois spots; merged with blue counterstain of cell nuclei by DAPI). The region of hippocampus of an AD model mouse (5 × FAD, [24]) is shown in comparison to a wild type mouse brain (Wt). (**B**) *E. coli* bacteria (DH5α) were grown for 48 h on YESCA agar and the biofilm stained with ThT (shown is a merge of bright field and green fluorescent channel).

Out of the known amyloidogenic proteins found in humans, 37 are correlated to diseases [5] such as systemic primary amyloidosis caused by monoclonal light chains (AL) or hereditary ALs such as those caused by ApoA1 or lysozyme [6,7]. While only about one third of all amyloidoses leads to proteinaceous deposits in the central nervous system, these diseases represent the most common forms of amyloid-derived disorders. AD and related dementias for example, affected more than 40 million people in 2016 [8] and there is an estimated number of more than six million PD patients world-wide [9]. The proteins contributing to these neurodegenerative diseases are well known and have been identified decades ago (see Table 1). For AD, APP gives rise—via subsequent proteolytic processing—to the amyloid-β peptide (Figure 1A) while in PD α-synuclein represents the amyloidogenic compound. For familial cases of both and other protein-deposit-based diseases, heredity of mutated variants of the amyloid-encoding genes or genes encoding respective processing enzymes are key to pathogenesis. The vast majority of cases still though is designated sporadic, indicating that the underlying mechanism has not been identified yet.

Progressively, relevance of microbial commensals came into focus as a possible contributing factor to neurodegenerative disorders (for concise reviews see [10,11,12]). Increasing number of reports provide correlative studies on altered microbiota in those kinds of diseases, but the outcome is quite controversial and evidence-based investigations are still scarce. Scheperjans and colleagues pinpointed this clearly by entitling a 2018 review with “The Gut and Parkinson’s Disease: Hype or Hope?” [13]. However, it seems that changes in at least some bacterial commensals have been consistently found. For PD, increased relative abundance of representatives from the genera Akkermansia, Lactobacillus, and Bifidobacterium and reduced abundances of Prevotella, Faecalibacterium, and Blautia have been shown [14,15,16,17,18]. For AD, Ruminococcus and S24-7 seem to be occasionally decreased while Odoribacter and Blautia as well as Alistipes have been shown to be increased in human and mouse model studies (summarized in [19]). Nevertheless, these are still only observational studies and lack functional conjunction between microbiota composition and pathogenesis. More recent investigations using e.g., fecal material transplants in mouse models might give deeper insight into mechanisms: the gut microbiota from a PD mouse model initiated motor impairment and decrease in striatal neurotransmitters in wild type mice while transfer of fecal material from wild type mice to PD mice ameliorated hallmarks of disease such as inflammatory status [20]. A daily application of fecal material from wild type mice was also found to reduce symptoms in the APPPS1 AD model mouse strain: performance in the Morris Water Maze task (a test reporting on learning and memory) increased significantly and amyloid-β brain levels were reduced by nearly 50% [21]. 

How the microorganisms can contribute to amelioration or aggravation of disease is still enigmatic. It is tempting to assume that bacterial metabolites such as short chain fatty acids are key players. Their effect has been proven in many studies (e.g., [22,23]). Another option is provided by the fact, that bacteria, fungi, and viruses are also capable of producing amyloidogenic proteins or peptides (see Figure 1B). Here in this review, focus was laid on the potential interplay of those microbial agents and their producers with the proteins and peptides underlying human neurodegenerative amyloidoses.

## 2. The Impact of Host Amyloidogenic Peptides on Microbial Commensals

Occurrence of bacteria in close surrounding to amyloid deposits in the brain of the human host has been demonstrated. For example, LPS and pili proteins of *E. coli* have been found in grey and white matter samples of Alzheimer’s disease patients while control samples remained negative in western blotting experiments [25]. Several periodontal pathogen spirochetes such as *Treponema medium* were additionally detected by PCR or antibodies or have been cultivated from AD brain material. For example, *Borrelia burgdorferi* was found in ¼ of all investigated AD cases (for an overview see [26]). For PD such data are—to this author’s knowledge—not available yet. However, especially for PD early α-synuclein deposits have been detected in the gut (for a critical review on the “gut first” hypothesis see: [27]). Moreover, APP positive staining and amyloid-β deposits have also been found in the gut of AD model mice and cases of human disease [28,29]. Therefore, a direct interaction between the amyloids derived by the host and those produced by bacterial commensals can be envisioned in different sites of the body.

The impact of human amyloidogenic peptides on microbes has been investigated so far for α-synuclein and amyloid-β peptides: they seem to act as antimicrobial peptides (AMPs) at least towards some microorganisms. Using recombinant α-synuclein-derived peptides of different lengths, a minimal inhibitory concentration (MIC) of 0.2 µM was found for tested bacteria (e.g., *E. coli*, *Staphylococcus aureus*) and MICs of 0.4 to 3.2 µM for fungal organisms such as *Candida albicans* [30]. A rhodamine-labelled peptide could be localized to *E. coli* cell membrane and *C. albicans* cytoplasm, indicating a direct way of interaction. For *E. coli*, a dose-dependent influx of a non-membrane-penetrant dye in cells with α-synuclein incubation was demonstrated, suggesting a membranolytic mechanism. Comparably, *Escherichia faecalis* and *S. aureus* also bound to and were agglutinated in the presence of amyloidogenic amyloid-β species (e.g., A-β X-42, [31]). Interestingly, the less toxic and less aggregation-promoting peptide species amyloid-β 1-40 and 2-40 did not reveal these properties. Moreover, the observed antimicrobial effect was also restricted to the highly amyloidogenic peptide variants. Additionally, incubation of *C. albicans* with homogenates from temporal lobe of AD cases resulted in reduced growth capability as compared to homogenates from healthy controls [32] while no difference occurred when administering cerebellar homogenates. The cerebellum is a tissue that is not majorly affected by degenerative processes in AD [33]. Together with the correlation of amyloid-β burden in tissue and growth reduction of the yeast cells, this underlines the antimicrobial property of the amyloidogenic peptide characteristic for AD.

Mice genetically modified to represent an aggressive familial onset type of AD (therefore also designated 5×FAD) were protected from *Salmonella typhimurium* infection after single intracranial injections [34]. This was demonstrated by reduced weight loss and bacterial brain load as compared to wild type controls. Similarly, *C. albicans* infections of *Caenorhabditis elegans* and cultured cell lines (H4-N or CHO-N) were ameliorated by amyloid-β peptides. Therefore, it has been speculated that the long time unidentified physiological function of the amyloidogenic peptides characteristic for neurodegenerative diseases might be found in antimicrobial defense (see for example [35]). Nowadays, with our high hygiene standards, this weapon is possibly backfiring. Prolonged life time resulting e.g., in leakiness of the blood brain barrier (e.g., [36]) together with reduced risk of infection might be causative to neurodegenerative diseases in the aging brain.

Among many open questions, future investigations will have to clarify if an altered microbial community as observed in AD and PD (e.g., [19,37]) will react in the same manner to the toxic peptides as compared to a “healthy” community or if an adaptation is achieved that lastly might explain the altered microbial composition. In vitro investigations lacking the physiological surrounding of e.g., the gut with all its gradients (surface—lumen; stomach—large intestine) and highly defined parameters such as oxygen availability might explain the correlations only to a minor extend.

## 3. Occurrence and Function of Amyloidogenic Peptides in Microbial Organisms

Several non-pathological amyloids have been described so far such as in melanosomes [38], hormone-derived amyloids to confer storage within pituitary gland [39] or CRES amyloids as a component of the normal epididymal luminal sperm maturation [40]. Therefore, amyloidogenic peptides are not only linked to human diseases but occur with a distinct physiological function also in healthy conditions.

**Figure 2:**
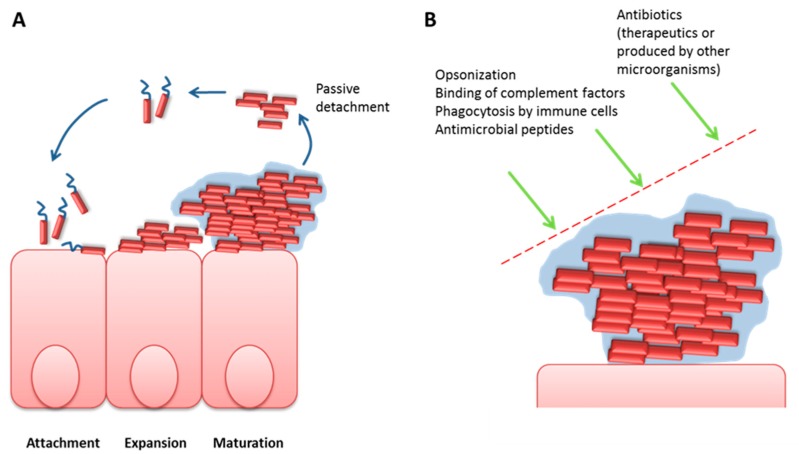
Biofilm formation on a biotic surface and protection against harms. (**A**) Bacterial biofilms form in three main steps: after initial attachment, bacteria expand and start to produce specialized components such as cellulose [45] to form a protective “slime” along with forfeiting motility. In a later phase, the mature biofilm allows detachment of clumps of cells or newly built cells to translocate to new areas of settling. Pore-building by PSMs (see Table 2) is assumed to play a major role in these events. (**B**) While one can imagine the biofilm to provide an ecosystem with restricted access to nutrition and space, it in contrast provides an especially protected area against attacks of the host immune system but also against exogenously added drugs or toxic compounds of other microbial species.

A recent review highlights the potential role of amyloids in the origin of life on Earth as these short aggregates are highly stable, can already form in prebiotic environment and might provide a basis for interaction with other polymeric structures such as RNA due to their repetitive character [41]. Therefore, their appearance in microbes, early settlers of our planet, seems highly plausible. Knowledge on their function is still restricted, however, when summarizing it, it seems that biofilm scaffolding and modification as well as attachment to membrane-surfaces are central (see Table 2). Biofilms are complex communities of bacteria grown on biotic (e.g., intestinal surface) or abiotic material (e.g., medical devices). When freely floating bacteria attach to surfaces, they start with a distinct expression program, resulting in production of exogenously deposited polysaccharides (firstly acknowledged by Costerton as biofilm-formation [42], Figure 2). They then also become progressively immobile (reviewed in [43]) and the surrounding matrix is completed by for example DNA (reviewed by [44]) and protein excretes such as amyloidogenic peptides.

The most intensely studied example of bacterial amyloids are the curli fibers found in *E. coli* which also have been found in Salmonella, Citrobacter, Enterobacter (for example [46,47,48]), and at least theoretically in Proteobacteria, Bacteroidetes, Firmicutes, and Thermodesulfobacteria (homologous encoding genes have been identified, [49]). In response to environmental conditions such as pH or nutritional sources, the master regulator CsgD drives expression of an operon encoding for CsgA and B (e.g., [50]). The protein CsgA aggregates with support from CsgB into curli fibers that can be stained by Thioflavin T or Congo red (see Figure 1, [51,52]) and support binding of the bacteria to different surfaces and molecules such as abiotic material or fibronectin [53,54,55].

*Staphylococcus epidermidis* and *S. aureus* are the predominant colonizers of medical implants in the clinic and *S. aureus* is the leading cause of morbidity and mortality among healthcare-acquired infections (e.g., Meticillin-resistant *S. aureus* (MRSA), [56]). From 94 cefoxitin-resistant *S. aureus* isolates derived from human patients, 65 were positive for a specialized phenol soluble moduline (PSM) which has a positive impact on biofilm formation [57]. The family of the PSMs contributes to various virulent properties (e.g., toxicity against human T-cells, [58]) and their various functions might be explained by different amyloid structures depending on truncation of the peptide [59].

Not only bacteria but also fungi and viruses have been shown to possess functional amyloidogenic proteins. Adhesin Als3, found in *C. albicans* cell wall for example, seems to have a pivotal role in developing communities with *Porphyromonas gingivalis* in the human oral cavity. This shifts gene expression of *P. gingivalis* into a more virulent pattern and thereby probably promotes inflammatory periodontal diseases [60]. The VP4 protease from avibirnavirus that causes immunosuppression and mortality in young chickens [61], was found to form tubular structures which on one hand prevent premature cell death of the host cells at the early disease stage but support cytoskeleton disarrangement at later stage [62]. This indicates that several microbial organisms use amyloidogenic peptides or proteins to regulate or achieve virulent properties (for an overview see Table 2) and leads to the question if and how such microbial components may influence the host’s own amyloidogenic structures.

## 4. Impact of Microbial Amyloids on Host Health and Neurodegeneration

Spreading of amyloidogenic material from a peripheral site such as the gut with its pleithora of commensals might not seem highly plausible at first sight. However, injection of AAV vectors encoding human α-synuclein in the left vagus nerve in the neck of rats sufficed to allow propagation to the pons, midbrain and forebrain [81]. Thioflavin-S positive stain thereby indicated amyloidogenic aggregation in a small number of neurites. The vagal nerve is one of the major connections of the gut-brain-axis. Interestingly, vagal gut–brain sensory (afferent) signaling was required for hippocampal-dependent learning processes as demonstrated by partially vagotomized rats in a modified Barnes maze [82]. Vagal afferent information is first received in the brain within the medial nucleus of the solitary tract which projects towards many brainstem and forebrain sites [83,84]. The finding that it also affects hippocampus without having direct connection envisions the opportunity that also other brain regions might depend on afferent vagus nerve input.

Autopsy studies suggested that α-synuclein-based Lewy body pathology initially occurs in the enteric nervous system (ENS) and from there proceeds to the dorsal motor nucleus of the vagus nerve. This was further supported by retrospective analysis of human patients which indicated that truncal vagotomy might be protective against PD [85]. By injecting pre-formed α-synuclein fibers into the mouse gastric wall, Lewy-body-like structures were obtained in the dorsal motor nucleus [86]. This could be prevented by vagotomy. Even if no further caudo-rostral propagation of amyloidogenic particles was observed, this investigation describes a putative first step in development of a neurodegenerative disease starting from the gut. Comparably, entry of orally ingested prion proteins from the gut with subsequent spreading to the brain is possible [87]. Interestingly, infection of local Peyer’s patches within the small intestine seems to be independent from endogenous cellular prion protein (PrP^c^) as shown in mice with epithelial deficiency of the protein in the lining of the small intestine [88]. Besides the vagus nerve, also other tissues might be implicated in further progression of amyloid spreading such as the olfactory epithelium, the mucosa of the oral cavity and the trigeminal nerve. Administering fluorescent amyloid-β peptides e.g., to the nostrils of mice after mannitol-based relaxation of the blood-brain-barrier led already after 1 h to signals in tissue homogenates derived from the hippocampus and cortical regions [89]. Moreover, this was accompanied by impaired learning and memory performance as measured by fear conditioning and Morris water maze task.

Besides the impact that bacteria might exert via pro-inflammatory potential versus the host, a direct influence of their amyloids, for instance derived by biofilm formation, is plausible. Direct evidence for human patients is—to my knowledge—still lacking. However, feeding of curli-producing *E. coli* to a *C. elegans* model with muscular expression of α-synuclein increased aggregation of these peptides in the worm [90]. Aged Fisher 344 rats exposed to the bacteria by gavage revealed induced aggregation of α-synuclein in the striatum and the substantia nigra accompanied by astrogliosis and microgliosis as compared to animals inoculated with vehicle or curli-deficient-bacteria. In a recent report, this was further substantiated by mono-colonization of α-synuclein overexpressing germ-free mice with curli-producing *E. coli* [91]. While colonization with a curli-deficient bacterial strain did not elicit motor impairment, beam traversal and pole descent were impaired in those animals inoculated with curli-producing *E. coli*. Similar results were obtained by administering the curli-producers on a complex fecal transplant background (human with low amount of curli-producers).

The N-terminal domain of HypF from *E. coli* (HypF-N) is also an amyloidogenic protein (see Table 2). Type A oligomers of HypF-N were found to colocalize with post-synaptic densities in primary rat hippocampal neurons [92]. Moreover, they inhibited CA1 hippocampal long-term potentiation in organotypic slices from rat. Intracerebral application of amyloid-β peptides in rats leads to impaired learning and memory as found in Alzheimer’s disease, e.g., assessed in the Morris water maze task (e.g., [93]). Interestingly, the intra-hippocampal injection of type A HypF-N oligomers in rats also resulted in similar memory impairment in the learning task [92], indicating that this bacterial amyloid can mimic synaptotoxicity of amyloid-β.

In sum, microbial amyloids seem to have the potential to affect human amyloid aggregation. If this holds true for a situation bearing physiological concentrations and parameters within the human body will surely be difficult to demonstrate.

## 5. Therapeutic Strategies Derived from Microbes against Human Amyloidosis

Probiotics have been suggested for therapeutic usage in regard to human neurodegenerative diseases such as AD. First reports indicate successful application in animal models: *Lactobacillus plantarum*, for example, was able to restore cognition and levels of acetylcholine esterase in the brain of a D-galactose-evoked AD-like rat model [94]. Similar results were obtained from investigations using genetic AD mouse models with *Bifidobacterium breve* strain A1 [95], SLAB51 (probiotic formulation, [96]) but also with *Lactobacillus acidophilus*, *Lactobacillus fermentum*, *Bifidobacterium lactis*, and *Bifidobacterium longum* in rodent sporadic AD models due to amyloid-β injection [97,98,99]. As only one explorative intervention study reports on a small cohort of AD human patients [100], the usefulness in humans is still not proven. Additionally, the underlying mechanism has not been resolved. However, amyloids from microorganisms also might be target to this treatment as *Lactobacillus helveticus* is for example able to produce biosurfactants that are able to suppress biofilm formation from *S. aureus* [101]. Moreover, a modified functional amyloid from Pseudomonas (FapC) not only itself showed prolonged lag time for aggregation but additionally attenuated α-synuclein fibrillation in vitro [102]. While the authors of this report themselves doubted that wild type FapC might have an impact on α-synuclein deposition in the gut due to its rapid self-aggregation, speculation about administering bacteria with such modified proteins might be nevertheless warranted. Additionally, significant amyloid release from *E. coli* biofilms upon induction by prophages has been described which might contribute to development of Type 1 diabetes-associated autoimmunity in children [103]. The stimulus leading to prophage induction has not been identified yet; however, this might open up new avenues for controlling curli-release also in people at risk for developing neurodegenerative diseases.

Interestingly, the green tea polyphenol epigallocatechin-3-gallate (EGCG), which has been tested as a therapeutic drug in AD and PD in animal models but also in patients [104], shows anti-amyloidogenic properties also on bacterial biofilms [105]. This indicates that these biofilms might also serve as simple models for identifying and investigating amyloid-destabilizing drugs for human use.

## 6. Conclusions

Potentially, many more amyloids will in future be identified in microorganisms. A proteomics approach in different yeast strains e.g., revealed 33 amyloid-like detergent-resistant proteins in *Saccharomyces cerevisiae* [106]. Moreover, further functional amyloids still may have to be identified in mammals (for a recent example: RNA-binding protein FXR1, [107]). Some of the microbial amyloid producers are physiological commensals that have a continuous impact on our health status and some might represent occasionally occurring pathogens that could promote amyloid deposition in the human body. As the human amyloids partially have been shown to act anti-microbial, future investigations will have to consider this as an additional aspect in unravelling the gut-microbiota-brain axis in human amyloidoses.

## Figures and Tables

**Table 1:** Human amyloidoses with manifestation in the central nervous system (neurodegenerative disorders). While some proteins/peptides represent a characteristic hallmark for just one disease, some are also found in various diseases such as hyperphosphorylated Tau protein.

Name of Peptide or Protein	Disease
α-synuclein	Parkinson’s disease (PD) Lewy body disease Multiple systemic atrophy
Amyloid-β	Alzheimer’s disease (AD)
Ataxin	Spirocerebellar ataxia
F-box protein 7 (FBXO7)	Parkinson’s disease (PD)/Alzheimer’s disease (AD)
Prion protein (PrP^sc^)	Transmissible spongiform encephalopathy (TSE)
Tau (hyperphosphorylated)	Frontotemporal dementia (FTD) Alzheimer’s disease (AD) Niemann Pick disease Progressive supranuclear palsy Amyotrophic lateral sclerosis (ALS)
Transactive response DNA binding protein 43 (TDP43)	Amyotrophic lateral sclerosis (ALS) Alzheimer’s disease (AD) Frontotemporal lobar degeneration with ubiquitin-positive inclusions (FTLD-U)
Superoxide dismutase 1 (SOD1)	Amyotrophic lateral sclerosis (ALS)
Huntingtin (with polyQ tract >33 residues)	Huntington’s disease

**Table 2:** Amyloidogenic proteins and peptides from microbial organisms. The indicated organism or family must not be imperatively the only organism producing the respective peptide or protein but was used in the underlying investigation. (nn: not known so far).

Name of Organism/Family	B = Bacterial F = Fungal V = Viral	Name of Peptide or Protein	Function	Reference
*B. burgdorferi*	B	Peptide designed from outer surface protein A (Osp)A	nn	[63]
*E. coli*	B	RNA binding protein Hfq	Interaction with biological membranes, potentially export of RNA	[64]
*E. coli*	B	MinE	Interaction with biological membranes, lipid redistribution	[65,66]
*E. coli*	B	Hydrogenase maturation factor HypF (HypF-N)	Permeabilization of membranes	[67]
Enterobacteriaceae	B	CsgB	Biofilm formation	[47,68]
*Gallibacterium anatis* *	B	Elongation factor-Tu (EF-Tu)	Adhesion-like function	[69]
*Mannheimia haemolytica*	B	Amyloid-like protein (ALP)	Cell adhesion, biofilm formation	[70]
*Mycobacterium tuberculosis*	B	Early secreted antigen 6-kDa protein (ESAT-6)	Potentially pore-formation	[71,72]
*Pseudomonas aeruginosa*	B	Functional Amyloid in Pseudomonas (Fap) C	Strengthening of biofilms	[73,74]
*S. aureus*	B	Phenol soluble modulins (PSMs)	Resistance of biofilms to various dispersion agents	[75]
*S. aureus*	B	N-terminal leader fragment of accessory gene regulatory (Agr) D	Seeding the amyloid polymerization of PSM peptides (in vitro)	[76]
*S. epidermidis*	B	C-repeat of Biofilm associated protein (Bap)	Potentially bacteria-bacteria-adhesion	[77]
*C. albicans*	F	Agglutinin-like sequence family 3 (Als3)	nn	[78]
*Saccharomyces cerevisiae*	F	glucantransferase Bgl2	Assumed cell protection against oxidative stress	[79]
*Avibirnavirus infectious bursal disease virus (IBDV)*	V	Viral protease VP4	Reduction of cytotoxicity of protease activity in host cells	[62]
Coronavirus	V	Peptide C6	nn	[80]

* Pathogenic in chicken.
